# Relationships between inflammatory and metabolic markers, exercise, and body composition in young individuals

**Published:** 2021-05-14

**Authors:** Sarah L. Dunn, Desiree L. Vera, Kathleen F. Weaver, Jerome V. Garcia

**Affiliations:** ^1^Department of Kinesiology, California State University, San Bernardino, Palm Desert Campus, Palm Desert, California; ^2^Department of Biology, University of La Verne, La Verne, California; ^3^Office of the Provost, Loyola Marymount University, Los Angeles, California

**Keywords:** interleukin-6, C-reactive protein, nuclear factor kappa B, cytokines, myokines, proinflammatory cytokines, anti-inflammatory cytokines, physical activity

## Abstract

**Background and Aims:**

Physical exercise may help combat disease and elicits a possible “protective” anti-inflammatory effect on the body. Inflammatory cytokines, C-reactive protein (CRP), interleukin-6 (IL-6), and tumor necrosis factor-α (TNFα), along with transcription factor, nuclear factor-kappa B (NF*κ*B) in young (n=16, 21.1±2.1 years) individuals were examined in a cross-sectional descriptive study, to assess the effects of chronic stimulation on their expression and relationship with health parameters.

**Methods:**

Fasting venous whole blood and lipid levels along with body composition measurements were obtained from young, healthy, endurance-trained NCAA Division III student-athletes and untrained individuals. Assays (ELISA) were conducted to analyze fasting plasma (CRP, IL-6, and TNFα) and isolated lymphocyte NF-*κ*B activation (lymphocytes were isolated from whole blood samples through differential centrifugation and Ficoll-Paque). A Spearman’s rank order correlation coefficient was used for associations between variables and a regression analysis was performed to determine which measurement accounted for the inflammation in this young and apparently healthy population.

**Results:**

While the inflammatory markers were not associated with each other, CRP levels were associated with body composition and following regression analyses, body fat percentage (*P*>0.05) was a significant factor for elevated CRP.

**Conclusions:**

Chronic physical exercise eliciting lower body fat percentages in young adults may have a positive protective impact through anti-inflammatory status, minimizing disease risk in a young population.

**Relevance for Patients:**

Chronic physically active young adult patients may exhibit less inflammation and lower body fat levels which may decrease their risk for chronic disease.

## 1. Introduction

Sedentary behaviors, aging, and the associated comorbidities may lead to an imbalance in metabolic and endocrine factors that have been triggered by underlying physiological stress and obesity [1,2]. A number of metabolic and endocrine factors play a role in bodily stress and inflammation, though recent studies concentrate on low grade systemic (chronic) inflammation linked to obesity, an excessive accumulation of adipose tissue [3], sex [4,5], aging, and other disease states [6-8]. A recent study in 2020 by Lee *et al*. shows a positive relationship between poor metabolic health and inflammation in adolescents [9]; however, overall, few studies have been performed with respect to younger populations free of disease states. Interleukin-6 (IL-6), C-reactive protein (CRP), tumor necrosis factor-α (TNFα), and the transcription factor, nuclear factor kappa B (NF-κB) have all gained attention as immune response markers, as they have been found to be elevated in an obese state [10].

### 1.1. Low-grade systemic inflammation

Inflammation is the body’s natural defense mechanism against infection and stress through the use of multifactorial cytokines [11]. Cytokines are protein signaling molecules that are released by cells and have the ability to alter the behavior of other cells as well as the releasing cell itself [11]. Circulating levels of cytokines are significantly affected by tissues outside of the immune system, such as adipose tissue and skeletal muscle (myokines) [12], enlightening obesity researchers to a greater understanding of the adipocyte and the role it plays in both chronic inflammation and to a greater extent, chronic disease. Adipose tissue is an important endocrine organ that stimulates the secretion of several pro-inflammatory cytokines (IL-6, TNFα, and CRP) [13] which, in obese individuals, has been shown to increase the accumulation of macrophages, providing a major cellular source of local TNFα expression [14]. An underlying mechanism for this inflammatory response is the transcription factor NF-κB, a central mediator of inflammation and stress responses, initiating transcription factors for inflammatory cytokines in adipocytes and myokines in skeletal tissue [15]; however, in the last year, it has also been labeled an age-related signaling up regulator for the inflammation cascade [8].

**Figure 1:**
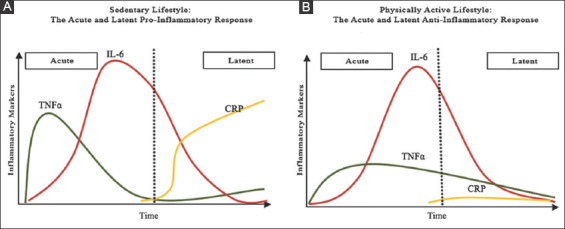
The acute and latent effect of a sedentary lifestyle (A) and physically active lifestyle (B) on the inflammatory markers interleukin-6, C-reactive protein, and tumor necrosis factor-α adapted from Petersen and Pedersen with permission [16].

According to Petersen and Pedersen [16], the inflammation cascade has an acute (within a few hours) and chronic (>8–12 h) response that results in the production of the inflammatory cytokines locally and systemically (Figure 1). While the acute phase should be short in duration, a sustained immune response with the release of the acute and chronic cytokines mentioned above is known as “low-grade systemic inflammation” and is associated with aging [17], obesity, hypothalamic dysfunction, type 2 diabetes mellitus (T2DM) [18], CVD [6], some forms of cancer [19], and most recently, the coronavirus SARS-CoV-2 [20,21]. Once the stimulus responsible for inducing the acute inflammatory response in low-grade systemic inflammation is diminished, the cytokines degrade, and the initial reaction ceases [16]. Following this event, CRP has a latent effect indicating chronic inflammation [22] due to its ability to stay in the circulation for an extended period of time. This phenomenon is summarized in Figure 1A and B, involving sedentary and more active lifestyles. Whether this low-grade state is an important indicator of disease risk in young, apparently healthy individuals is unknown.

### 1.2. Exercise and inflammation

Similar to aging and obesity, exercise can cause both physical and physiological stress in the body. However, while initial physical exercise may induce an acute phase response [17] if the physical exercise is sustained over time (lifetime behavior), studies have found positive health benefits [23], including the ability to initiate an anti-inflammatory state through sustained muscle contractions. For example, studies have shown that regular exercise greatly decreases the concentration of inflammatory cytokines [24,25] while reducing the need for the hormone insulin to control blood glucose as muscle contractions uptake glucose [26]. A 2020 animal study by Wang *et al*. found a controlled diet and swimming exercise-induced positive benefits on the hypothalamus, which could greatly influence metabolic health, aging, and obesity [18]. What sets physical exercise stress apart from other inflammatory-inducing reactions is the release of the pro-inflammatory cytokine and anti-inflammatory myokine, IL-6, which is activated through muscle contractions [27,28]. It is important to note that IL-6 is synthesized and released only from contracting (not resting) muscle tissue as an anti-inflammatory mediator [16].

The IL-6 myokines contain binding sites for NF-κB, which may contribute to the activation of IL-6 gene transcription during physical exercise in an anti-inflammatory manner [15]. NF-κB is now identified as a key mediator of inflammation since it regulates cytokine and immunoglobulin gene expression [29]. Nevertheless, physical exercise-induced activation of NF-κB, leading to the secretion of IL-6 from contracting skeletal muscle, has been shown to decrease elevated plasma concentrations of TNFα and eventually CRP. Through the release of cytokine inhibitors and anti-inflammatory myokines [27] this feedback loop leads to a decrease in low-grade systemic inflammation. Adding to the depth of the IL-6 cascade signaling chain of events, elevations in IL-6 from contracting muscles have been noted to increase insulin sensitivity and possibly decrease ones risk of developing insulin resistance [30], the precursor for T2DM. Overall, multiple studies have concluded that lifelong physical exercise carries greater health benefits in its promotion of anti-inflammatory myokines and increasing insulin sensitivity [7,31]. With the ongoing elevated levels of IL-6 from contracting muscles, signaling cascades have been noted to stimulate lipolysis, in conjunction with catecholamine and cortisol levels [28], carrying the benefit of exercise into adipocyte degradation and ultimately the reduction of obesity.

While a multitude of studies address exercise protocols and the health benefits associated with physical exercise, the focus has mainly been on unhealthy or older populations [7,17]. The effects of physical exercise on the metabolic profile and inflammatory state of young, healthy individuals are relatively unknown. This study aimed to examine young trained and untrained, healthy individuals’ body composition (body mass index [BMI] and fat mass), blood plasma lipids (triglycerides [TRG] ad low-density lipoproteins [LDL]), inflammatory markers (IL-6, TNFα, and CRP), and the transcription factor NF-κB. The main hypothesis of this cross-sectional descriptive study was that young, untrained individuals at risk for obesity and aging-related factors would reveal a more chronic inflammatory status (correlating with molecular expression) compared to their young, healthy trained counterparts. The findings ultimately demonstrate the significant and positive impact of physical exercise on inflammation and body composition.

## 2. Methods

### 2.1. Participants

After obtaining Institutional Review Board (IRB) approval, young, apparently healthy participants (age: 21.1±2.1 years) were recruited from a small private university in Southern California and screened to determine their physical exercise status. Participants were either inactive (completing 2 h or less of physical exercise per week) or active through endurance training (seasoned NCAA Division III student-athletes in track and field distance events and cross country) to assess a varied population. After signing an IRB-approved informed consent form, participants were asked to visit the University Kinesiology Laboratory for two fasting (12 h) sessions and one visit to the Clinical Laboratory for a fasting venous blood draw performed by a trained phlebotomist. Participants were advised not to exercise 24 h before the venipuncture draw, so any variations observed in inflammatory markers would not be associated with recent physical exercise. The study took place immediately following the peak of the athletes’ cross country and track and field season(s).

### 2.2. Body composition and blood pressure

Height (HT), body mass (M), waist circumference (WC), hip circumference (HC), percent body fat (PBF), fat mass (FM), fat-free mass (FFM), and BMI were measured using a standardized tape measure, stadiometer (Seca 231, USA), and a bioelectrical impedance analysis scale (Tanita 310, Japan). Female participants’ menstrual cycle phase was obtained, and all-female participants were asked to return for post-data collection at the same menstrual phase as pre-data collection. Participants WC (cm) was measured between the lower margin of the last palpable rib and the top of the iliac crest and HC (cm) around the widest portion of the gluteal muscles. BMI was calculated by dividing mass in kilograms by height in meters squared (kg/m^2^). Resting systolic and diastolic blood pressure (SBP and DBP) were measured (average of two readings, 1 week apart) using a manual sphygmomanometer (LifeSource A and D, USA) and stethoscope (Littman, USA), and all measures were assessed in a 12-h fasting state between 7 am and 11 am.

### 2.3. Blood specimens

A fasting lipid panel (TRG, LDL, high-density lipoprotein [HDL], total cholesterol [TC], and glucose) assessment was completed using a finger prick for whole blood analysis (Alere Cholestech LDX, USA). All lipid values were compared to a clinical definition of a healthy range [32]. A trained phlebotomist drew a 40 mL fasting whole blood sample, which was transported back to the University Biology Laboratory for further processing. All blood samples were centrifuged (Eppendorf 5810 R) at 1500 rotations/min for 10 min at 4°C. Immediately following centrifugation, plasma was placed into 1mL micro-tubes and frozen (−80°C) for later analysis, while the buffy coat was used for the isolation of the primary blood mononuclear cells to determine the NF-κB subunits (p-50 and p-65).

### 2.4. Peripheral blood mononuclear cell isolation for NF-κB analyses

The buffy coat of each whole blood centrifuged sample was removed, and cells were washed with phosphate-buffered saline (HyClone PBS, Fisher Scientific). Differential centrifugation with the use of Ficoll-Paque premium (GE Healthcare, Fisher Scientific) was used to isolate lymphocyte populations at 1500 g for 15 min at 20°C (Eppendorf 5810R). After separation, lymphocytes were washed twice before culture, first with PBS, then with Roswell Park Memorial Institute media (RPMI-1640 Medium, Sigma Aldrich). Lymphocytes were then cultured for 24 h in RMPI with 10% fetal bovine serum at 37°C with 5% CO_2_ and 95% Relative Humidity (Model SCO_2_W, Sheldon Manufacturing Inc., USA). After culture, suspended lymphocytes were removed and then washed and brought to a dry pellet. Dry pellets were stored at −80°C for later analysis.

### 2.5. Immunoassays

Plasma samples were thawed and analyzed in duplicate using enzyme-linked immunosorbent assays (ELISA) specifically designed and validated for the quantitative measurement of human blood plasma insulin, high sensitivity IL-6, TNFα, (RnDSystems, USA), and high sensitivity CRP (Calbiotech, USA). The range and coefficient of variability for each ELISA were as follows; insulin (2.25–72 μg/dL, 3.6%), HS-IL-6 (0–10 pg/mL, 6.5%), TNFα (0–1000 pg/mL, 6.2%), and HS-CRP (0.0005–0.1 mg/L, 7.9%).

Isolated lymphocytes were also thawed, and protein determination was performed to define the lymphocyte volume for further analysis. A p50 and p65 Colorimetric ELISA (Signosis, USA) kit to quantitatively measure activated NF-κB protein dimmers was used based on the resulting volume from the protein determination. The coefficient of variability for the ELISA was CV 20%.

### 2.6. Statistical analysis

IBM SPSS Statistics software, version 21, was utilized for descriptive statistics (mean±standard deviation) and inferential statistics for all variables. To determine statistically significant relationships (*P*<0.05), Spearman’s Rank Order Correlations were run to find the associations between inflammatory markers and other variables measured across all subjects and samples. A General Linear Model analysis [33] was performed to examine influential variables on the inflammatory markers.

## 3. Results

### 3.1. Overall population

Sixteen fasting males and females completed all testing sessions. Overall participant characteristics and group variables are presented in Table 1. All participants were considered healthy and did not meet the criteria for metabolic syndrome or T2DM at study initiation.

**Table 1:** Mean study population characteristics

	All (n=16) Female 69% Male 31%	Trained (n=7) Female 43% Male 57%	Untrained (n=9) Female 89% Male 11%
**Parameters**	**Mean±SD**	**Mean±SD**	**Mean±SD**

Age (years)	21.1±2.1	20.7±1.7	21.4±2.4
PBF (%)	24.9±13.8	12.0±6.5	34.8±8.3
BMI (kg/m^2^)	24.6±±5.4	20.8±1.9	27.5±5.4
WC (cm)	76.7±14.2	68.9±6.2	82.7±15.9
FM (kg)	18.6±13.5	7.1±2.9	27.5±11.3
FFM (kg)	51.8±12.7	54.9±12.1	49.4±13.4
Glucose (mg/dL)	90.9±7.7	89.1±5.1	92.2±9.3
TRG (mg/dL)	139.9±55.1	69.3±9.9	171.3±30.5
HDL (mg/dL)	55.9±12.0	61.9±9.9	50.6±11.8
LDL (mg/dL)	92.9±13.0	93.8±7.3	92.5±15.6
SBP (mmHG)	99.9±12.0	100.0±5.6	99.8±15.7
DBP (mmHG)	62.7±7.6	64.5±7.8	61.2±7.6
Insulin (µg/dL)	5.0±3.5	4.0±0.47	5.7±4.6
CRP (mg/L)	0.03±0.03	0.01±0.01	0.05±0.03
IL-6 (pg/mL)	2.3±1.2	2.0±0.8	2.5±1.3
p50 subunits	0.21±0.05	0.22±0.05	0.20±0.05
p65 subunits	0.16±0.04	0.17±0.05	0.16±0.04
TNFα (pg/mL)	1.2±0.5	1.2±0.6	1.2±0.5

### 3.2. Body composition, insulin, and TRG

FM was positively associated with fasting plasma TRG (*rho*=0.731, *P*<0.05) and negatively associated with fasting plasma HDL levels (*rho*=−0.707, *P*<0.05). A significant relationship was found between insulin and fasting plasma TRG levels (*rho*=0.593, *P*<0.05).

### 3.3. Relationships between inflammation and other variables measured

HS-CRP was significantly associated with body composition measures (FM, *rho*=0.700, *P*<0.05, PBF, *rho*=0.632, *P*<0.05, and BMI, *rho*=0.643, *P*<0.05). A trend toward significance was found between HS-CRP and IL-6 (*rho*=0.486, *P*=0.08). No relationship was found between the acute inflammatory markers and the NF-κB subunits p-50 or p-65 in this population. The NF-κB subunits p-50 or p-65 were significantly related (r=0.853, *P*<0.05) and a trend between glucose and the p-65 NF-κB subunit was noted (r=0.466, *P*=0.07). PBF showed to be a significant (*P*<0.05) predictor of HS-CRP apart from all other variables measured. No significant predictors were found following the regression analysis for the acute inflammatory markers and the NF-κB subunits p-50 or p-65. When males were removed from the analysis to assess for gender differences, only the associations between CRP and BMI (*rho*=0.673, *P*<0.05, n=11) and CRP and FM (*rho*=0.609, *P*<0.05, n=11) remained significant; all others were not significant.

## 4. Discussion

Several studies in the literature document the significant role of excess adipose tissue and the influence it has on the inflammatory markers, IL-6, TNFα, and CRP [34], although the individuals are usually categorized as unhealthy (e.g. obese, diabetic) and aged [17]. As mentioned previously, a recent study by Lee *et al*. (2020) found associations between metabolic markers and inflammation in adolescents age 13–15 years from the Ewha Birth and Growth Cohort Study [9]. More studies involving young and apparently healthy populations focusing on health and inflammation parameters while stratifying for gender, especially for those that are engaged in physical exercise, are needed. The new national prescription to the obesity epidemic is a preventative and educational approach to a younger generation and with the current COVID-19 pandemic, overall health is a concern for many globally. This study set out to assess the relationships between inflammatory markers, NF kappa B subunits, and metabolic markers, including body composition in young active and inactive individuals.

### 4.1. Summary of participant data

Due to the study’s low sample size, differences between trained and untrained as well as between genders within the groups could not be statistically evaluated for any of the markers measured. Gender may have had an impact on the inflammation status in the current study as the untrained group had more females compared to the trained group. The current literature indicates young or overweight/obese females may have elevated inflammatory cytokines or lower anti-inflammatory markers [4,5] due to the role of the hypothalamic-pituitary-adrenal axis and sex hormones and/or involving low adiponectin (anti-inflammatory) levels. The greater number of females in the untrained group may have influenced the increased levels of inflammation, although the sample is small for the current cohort and did not match up for all relationships found when males were removed. Correlations in the female sample were only found with CRP and BMI and FM, but not PBF. When males were removed from the analysis, all other variables were found to be non-significant. The influence of the female menstrual cycle was not evaluated for any of the variables measured and may have had an impact on the variables assessed.

Even in a healthy state, the integration of routine physical exercise brings about metabolic benefits, and this study highlights the impact of an active lifestyle at a young age for the prevention of disease. Physically, the untrained group is not considered to be clinically unhealthy which is explained in greater detail in the subsequent paragraphs; however, their chronic inflammatory status (elevated HS-CRP levels) along with elevated TRG and adiposity increases their risk for disease. A prescription of physical exercise for the untrained group may activate IL-6 as an anti-inflammatory myokine, ultimately leading to lower CRP levels and regulating glucose uptake into the muscle positively influencing glycemic control, along with a reduction in adipose tissue and TRG levels, which in the long term will potentially decrease the risk of disease.

### 4.2. Physical exercise, body composition, and lipids

Body composition (FM) and lipids (TRG) had a significant positive relationship, which researchers have reported previously [35]. The untrained population in the current study had an average TRG level of 171.33±30.55 mg/dL, which is within the borderline high category (150–199 mg/dL), considering this is a younger (healthy) population, these levels increase their risk for disease [36]. Although TRG levels reflect the amount of fat in the blood and are greatly influenced by dietary intake [37], Tsekouras *et al*. [38] found a 24% reduction in very low-density level (VLDL) TRG concentration 12 h after brisk walking (60% of VO_2_ max for 90 min) with a 35% increase in VLDL-TRG clearance in young, healthy men. This solidifies the argument that even moderate exercise (i.e. walking versus running) will positively affect the blood lipids and therefore may influence body composition and eventually inflammation in a young population.

### 4.3. Physical exercise, insulin, and acute inflammatory markers

Given the fact that the study population consisted of young healthy adults, it was hypothesized that a relationship in inflammation would be found based on adipose levels. According to Matsusaka *et al*. [15], an excessive amount of adipose tissue may alter gene expression of NF-κB, leading to a signaling cascade, increasing IL-6 (pro-inflammatory), TNF-α, and eventually CRP levels. All levels in the acute inflammatory markers (IL-6, TNFα) and gene expression (NF-κB) subunits were within a healthy range (Table 1) and while IL-6 and TNF-α were significantly related (*P*<0.05) to each other, a trend toward significance was found in the relationships between the acute (IL-6) and chronic inflammatory (CRP) markers (*P***=**0.08). These results verify the pro- and anti-inflammatory actions of IL-6 and the relationship it has with physical exercise, in addition to the relationship between acute and chronic inflammatory markers. The endurance-trained individuals participated in daily sessions of physical exercise, suggesting that the IL-6 value is predominantly caused by exercise-induced IL-6 secretion (in an anti-inflammatory capacity). Contracting muscle fibers produce and release IL-6, which induces several positive metabolic effects, such as the regulation of TNF-α (pro-inflammatory) levels [25] and increased glucose uptake in the contracting skeletal muscles [39]. As was seen in 2013, no differences were found between IL-6 and TNF-α levels between elite kayakers and non-athletes [40] which coincides with the current findings.

In the case of NF-κB levels and exercise, this transcription factor stimulates the production of IL-6 and is redox-sensitive, which may be activated by redox sensitive species produced by exercising skeletal muscles [41] and could have contributed to this study’s NF-κB results. While it is concluded that IL-6 and NF-κB values in the trained group are influenced by contracting muscles, the untrained groups’ IL-6 and NF-κB values cannot be explained by physical exercise alone. Interestingly, the positive relationship found between IL-6 and insulin may help reveal the answer in the untrained population. A lack of routine physical exercise may lead to a decrease in insulin sensitivity resulting in insulin resistance, as shown by two different studies conducted by Ross *et al*. and Christ-Roberts *et al*. in 2004 [42,43]. Those individuals in the current study with elevated insulin levels were found to have higher acute inflammatory markers, which was not the case for HS-CRP. Therefore, those with the early signs of metabolic dysfunction (elevated fasting plasma insulin) most likely are acutely inflamed, whereas those with increased adiposity (untrained) are associated with chronic inflammation (elevated HS-CRP). For example, Adams *et al*. [44] found that in a population of obese African Americans, those who participated in >60 min/day of physical exercise had 62% lower CRP levels than their sedentary counterparts. Dutra *et al*. [45] also showed that a significant gain in FM (central obesity) and a significant loss in FFM may be a contributing factor in high concentrations of CRP in post-menopausal women. While endurance training may protect healthy young individuals from inflammation (acute and chronic), a lack of physical exercise may lead to either metabolic perturbations or excess adiposity, and both may lead to an inflamed state, either acute or chronic, increasing one’s risk for disease.

### 4.4. Physical exercise, body composition, and chronic inflammatory markers

Based on the data, the trained individuals’ IL-6 levels were related to their physical exercise status instead of metabolic markers or adiposity levels. The untrained group had significantly elevated HS-CRP (chronic inflammatory) levels (5 times greater) compared to the trained individuals, further supporting that physical exercise protects young, healthy subjects from acute to chronic inflammation. While CRP secretion increases following IL-6 secretion from macrophages, T-cells, and adipocytes [46], the levels are not elevated from contracting muscles. Further research on the physiological mechanism behind physical exercise lowering CRP levels (similar to what is found with the acute inflammatory cytokines) is needed as it is not well defined in the literature at this time, for any age. We did find a positive correlation between BMI and HS-CRP levels, concordant with the results of [3] from a subject age range of 17–39 years old. In addition, in the present study, HS-CRP was linked to FM in this young trained and untrained group and following regression analysis, body fat percentage was the sole significant predictor of elevated HS-CRP. The excess adipose tissue is responsible for the increased secretion of IL-6 (pro-inflammatory) and TNFα, without any relief from the anti-inflammatory benefits of exercise, therefore increasing the secretion of CRP.

Future studies may need to assess the validity of CRP as a long-term biomarker for chronic inflammation as it may be influenced by a variety of factors (environmental, metabolic, adiposity, etc.). Finally, no standard value has been determined as of today for the inflammatory markers mentioned here; establishing “healthy” levels for each inflammatory marker within a young healthy population may verify if these participants were within the normal range despite other metabolic perturbations, training status, or adiposity.

In conclusion, physical exercise, a type of physiological stress placed on the body, may elicit pro-inflammatory cytokines initially, although chronic physical exercise may offer protection against low-grade systemic inflammation through muscle contraction-induced anti-inflammatory properties and reductions in adiposity. Adiposity (body fat percentage) was the best predictor of elevated HS-CRP in this population. These cross-sectional findings in young adults allude to the benefits of maintaining physical exercise in young adulthood, which may combat low-grade systemic inflammation, ultimately improving disease risk.
